# Injectable Colloidal Hydrogels of *N*-Vinylformamide Microgels Dispersed in Covalently Interlinked pH-Responsive Methacrylic Acid-Based Microgels

**DOI:** 10.1021/acs.biomac.3c00058

**Published:** 2023-04-07

**Authors:** Xuelian Wang, Daman J. Adlam, Ran Wang, Amal Altujjar, Zhenyu Jia, Jennifer M. Saunders, Judith A. Hoyland, Nischal Rai, Brian R. Saunders

**Affiliations:** † School of Materials, University of Manchester, MECD Building A, Manchester M1 7HL, U.K.; ‡ Division of Cell Matrix Biology and Regenerative Medicine, The University of Manchester, Oxford Road, Manchester M13 9PT, U.K.

## Abstract

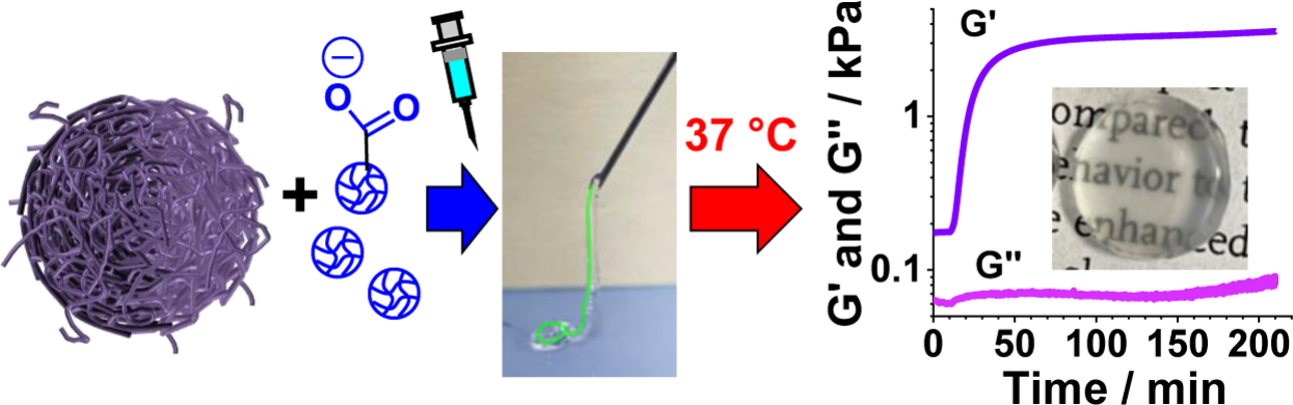

Injectable hydrogels offer great potential to augment damaged or degenerated soft tissues. A key criterion for such gels is that their modulus is as close as possible to that of the target tissue. The majority of synthetic hydrogels have used low molecular weight polymer chains which may cause problems if they diffuse away from the injection site and/or increase the local osmotic pressure. We previously introduced a different approach of injecting preformed ultra-high molecular weight pH-responsive microgels (MGs) that interlink to form hydrogels. MGs are crosslinked polymer colloid particles that swell when the pH approaches the particle p*K*_a_. These colloidal hydrogels are termed doubly crosslinked microgels (DX MGs). The gel moduli of previous DX MGs were much greater than that reported for human nucleus pulposus (NP) tissue of the spinal intervertebral disk. Here, we replace some of the pH-responsive poly(ethyl acrylate-*co*-methacrylic acid) (PEA-MAA) MGs with hydrophilic non-ionic MGs based on poly(*N*-vinylformamide) (NVF). We investigate the morphology and mechanical properties of these new injectable composite DX MGs and show that the mechanical properties can be tuned by systematically varying the NVF MG content. Using this approach, the gel moduli close to that for NP tissue are achieved. These injectable new pH-responsive gels exhibit low cytotoxicity. Our work provides a potential new system for minimally invasive intervertebral disk augmentation.

## Introduction

Degeneration of the intervertebral disk (DIVD) is the leading cause of low back pain and causes a major negative societal and economic impact. It is the second most common cause of adult disability in the United States^1^ and has become the top cause of loss of quality-adjusted life years.^2^ Low back pain causes more global disability than any other condition and is prevalent in aging populations and those with highest life expectancies.^3^ Currently, the gold standard treatment for DIVD is invasive spinal fusion, which is not effective in the long term.^4,5^ DIVD causes fissures in the load-bearing nucleus pulposus (NP).^6^ Such tissue defects are an ideal target for minimally invasive injectable gels.^7−11^ Injectable gels offer the advantages that they can reach and fill deep defects and do not require invasive surgery.^12−15^ We previously established injectable doubly crosslinked microgels (DX MGs) as a potential approach for IVD repair.^16^ Microgels (MGs) are crosslinked polymer colloid particles^17−20^ that swell when the pH exceeds the p*K*_a_ value of the particles.^21,22^ DX MG hydrogels are composed of covalently interlinked MGs^23^ and are a type of colloidal hydrogels.^9^ These hydrogels differ from injectable gel composites that rely on non-covalent inter-particle interactions for gel formation.^24−26^ However, the modulus of our previous DX MGs is higher than that reported for the human NP tissue^27^ which may adversely affect the IVD function. Here, we introduce a new injectable composite DX MG that enables the modulus of injectable DX MGs to be tuned to that of the NP tissue and also increases gel ductility.

There are a number of gels with excellent mechanical properties such as double-network gels,^28,29^ nanocomposite gels,^30,31^ and dynamic gels.^32−34^ Unfortunately, the requirement for injectability places severe constraints on the chemistry that can be used for in vivo gel formation.^1^ For example, assembly of small molecules (e.g., through polymerization) may be problematic as such species could migrate away from the injection site prior to gel formation. For a DIVD treatment, the injectable gels should mimic the NP tissue. The latter consists of a network of highly hydrated sulfated glycosaminoglycans which provide the high swelling pressures responsible for load support.^6,35^ Gels based on poly(*N*-isopropylacrylamide),^36,37^ chitosan,^38^ alginate,^39^ and cellulose^40^ have been investigated for IVD repair. Unfortunately, a clinically approved injectable gel for NP augmentation and/or repair is still elusive. A key challenge for injectable gels for IVD repair is that they should provide load support upon injection. High concentrations of ionic groups within the gel matrix can provide a high swelling pressure^41^ and, in principle, load support. We achieve that goal in the present study using pH-responsive MGs with high methacrylic acid (MAA) contents. Another criterion for an injectable gel is that the modulus should be close to, or ideally matching, that of the target tissue.^42^ In this study, we achieve this by blending two types of MGs to form a new type of injectable DX MG composite.

An ideal injectable gel is shear thinning, allowing injection, and rapidly forms a stable gel after filling the defect.^8,13^ Uniquely, DX MGs use pH-responsive vinyl-functionalized MG particles as colloidal-scale macrocrosslinkers. Our MGs contain a high content of MAA and swell when the pH increases to physiological pH (which is above the particle p*K*_a_ value), transforming the low-viscosity injectable fluid into a physical gel of ionic MGs which fill defects.^16^ The physical gel forms because the swollen MGs occupy the whole space of the mixture which prevents translocation of the particles. In the presence of an initiator and an accelerator, the physical gel transforms to a chemical gel of covalently interlinked MGs at 37 °C.^16^ This occurs by the free-radical reaction of vinyl groups from neighboring MGs that are sufficiently close together due to interpenetration of MG peripheries. The free-radical chemistry employed for DX MGs is similar to that used for bone cement. However, our DX MG approach avoids the use of small molecules because the overwhelming majority of gel assembly is conducted prior to injection.

**Scheme 1:**
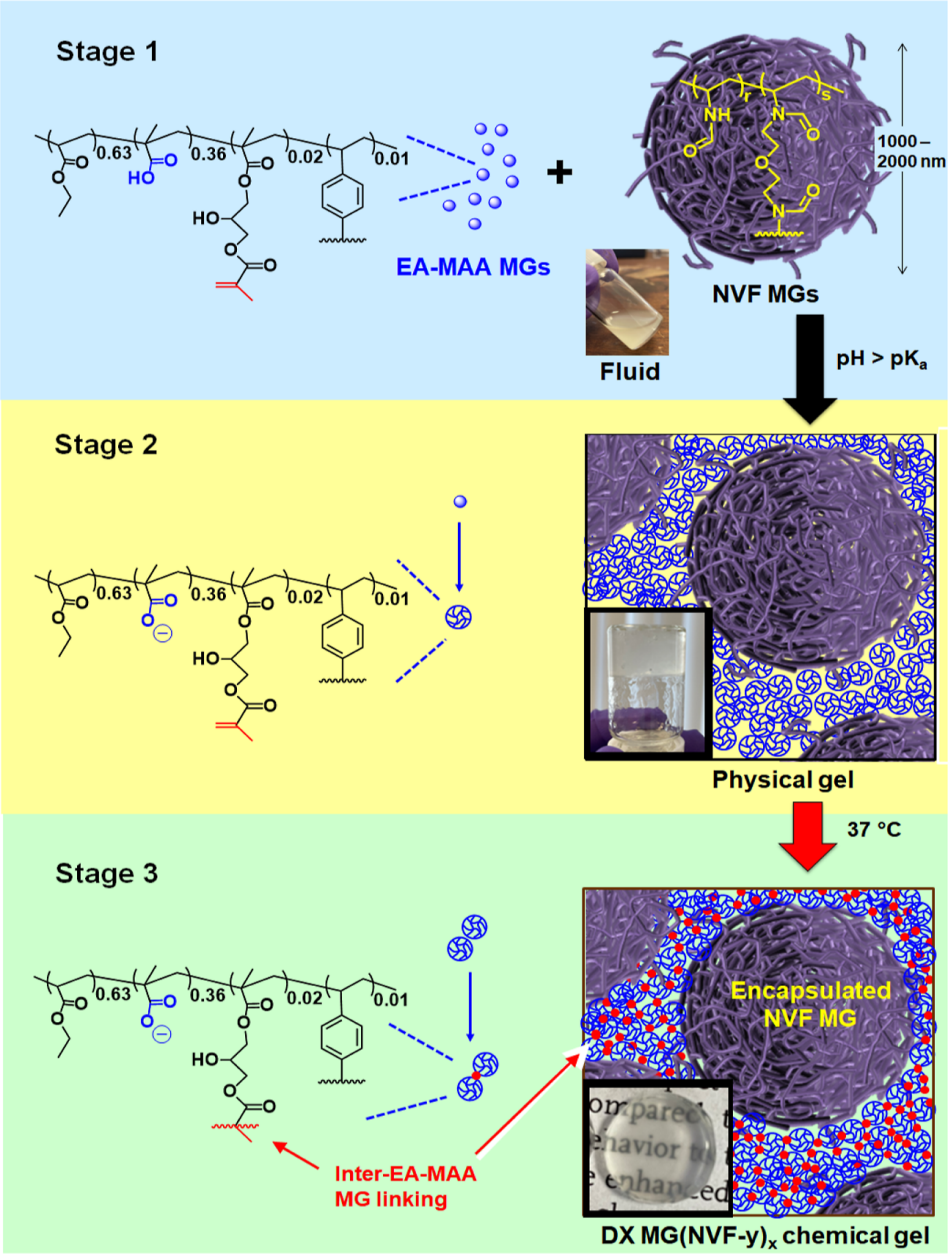
Depiction of the Formation of DX MG(NVF-*y*)_*x*_ Hydrogels where *x* and *y* Are the NVF MG Concentration Used (in wt %) and the NVF MG Type, Respectively; the Process Occurs in One Step and the Three Stages of Gel Formation Are Depicted

DX MGs have been used to establish high-modulus composites with graphene oxide^43^ or electrically conducting gels with carbon nanotubes.^44^ We reported that the binary blends of vinyl-functionalized MGs where one MG contained relatively long spacers connecting the vinyl groups to the MGs gave DX MGs with modulus values that could be varied.^45^ However, the synthesis of the MGs containing long spacers required the use of highly toxic epichlorohydrin, which may prohibit future biomaterial application. Here, we adopt a different approach and incorporate non-ionic, hydrophilic, poly(*N*-vinyl formamide) (PNVF) MGs into DX MGs for the first time (Scheme 1). The gels are termed DX MG(NVF-*y*)_*x*_, where *x* and *y* are the NVF MG concentration and the NVF MG type, respectively. The non-ionic nature of NVF MGs was expected to result in relatively low swelling pressures and softer MGs compared to the ionic MAA-containing MGs used to construct the DX MG matrix. NVF shows highly favorable toxicological properties^46^ and has good potential to replace its structural isomer, acrylamide, in many applications.^47^ PNVF is a biocompatible polymer^48^ that has been prepared as micrometer-sized particles.^49^ We hypothesized that inclusion of soft non-ionic PNVF MGs would provide a potentially useful method for decreasing DX MG modulus for future IVD repair. Furthermore, the inclusion of micrometer-sized PNVF MG particles was anticipated to enable their distribution within the DX MG matrix to be conveniently studied.

We prepare new composite DX MGs by blending two MGs with different properties and diameters: ethyl acrylate (EA)-MAA MGs and NVF-MGs (see Scheme 1). EA-MAA nanoparticles have been successfully used within in vivo studies for a diabetes therapy.^50^ Our EA-MAA MGs are pH responsive and have sub-micrometer diameters. They are also vinyl-functionalized and form inter-MG networks (DX MGs) when free radicals are present. The uncharged NVF MGs have supramicrometer diameters, are not vinyl-functionalized, and are not capable of forming DX MGs. We show that the NVF MGs are encapsulated within the DX MG networks as depicted in Scheme 1. We first examine the properties of the MGs and then study the morphology and mechanical properties of the composite DX MGs. We increase the proportion of NVF MGs in the composite gels, which decreases the modulus to values that approach that of human NPs. After investigating their pH-triggered swelling behavior, we examine the injectability and cytotoxicity of the gels. This study demonstrates a useful method for tuning DX MG mechanical properties and provides new injectable composite gels that have the potential for future use in IVD augmentation.

## Materials and Methods

### Materials

NVF (98%), potassium *tert*-butoxide (PTB, 95%), bis(2-bromoethyl) ether (BBE, 95%), dicyclohexyl-18-crown-6 (98%), anhydrous tetrahydrofuran (THF, 99.9%), chloroform (CHCl_3_, 98%), ethanol (99.9%), azoisobutyronitrile (AIBN, 98%), and poly(1-vinyl-pyrrolidone-*co*-vinyl acetate) (poly(VP-*co*-VA)) (average *M*_w_ ∼ 50,000) were all purchased from Aldrich and used as received. EA (99%), MAA (99%), glycidyl methacrylate (GMA, 97%), divinylbenzene (DVB, 80%), NaOH (97%), ammonium persulfate (APS, 98%), sodium dodecyl sulfate (SDS), *N*,*N*,*N*′,*N*′-tetramethyl ethylenediamine (TEMED, 99%), dipotassium phosphate (K_2_HPO_4_, 97%), and methylene violet (3RAX) were also purchased from Aldrich and used as received. All water was of ultra-high-purity deionized quality.

### NVEE Synthesis

2-(*N*-Vinylformamido)ethyl ether (NVEE) is the crosslinker for the NVF MGs. The synthesis for NVEE was described in detail earlier.^49^ Briefly, a comonomer containing NVF (7.1 g, 100 mmol), PTB (12.0 g, 105 mmol), dicyclohexyl-18-crown-6 (1.00 g, 2.65 mmol), and anhydrous THF (100 mL) was added to a reactor under mechanical stirring. The mixture was stirred vigorously for 45 min at room temperature and then cooled to 0 °C using an ice bath. BBE (9.30 g, 40 mmol) was added to the reaction flask dropwise. The mixture was stirred at room temperature for an extra 72 h. KBr was removed by filtration, and THF was removed by rotary evaporation. The product was diluted with water (100 mL) and washed with CHCl_3_ (50 mL) five times and concentrated aqueous NaCl solution (23.3 wt %, 100 mL) three times. The product obtained was dried over anhydrous sodium sulfate for 24 h. The residual CHCl_3_ was removed by rotary evaporation. NVEE was a liquid (5.69 g, 82% yield) with a purity of 83% as determined by ^1^H NMR spectra (see Figure S1). The liquid had a light brown color.

### NVF MG Synthesis

The synthesis route for the NVF MGs was described earlier^2^ and is depicted in Scheme S1. Here, the size of the NVF MGs varied by increasing the NVF concentration during the synthesis. Four NVF MGs were synthesized (termed NVF-A to NVF-D) by non-aqueous dispersion polymerization in latex form as non-swollen particles dispersed in ethanol. This study mostly focusses on NVF-A and NVF-D. (The key characterization parameters for all of the MGs used in this study appear in Table S1.) The method described below applies for all of the NVF MGs. The only difference between the various NVF MG syntheses is the mass of NVF added. The masses of the latter used for NVF-A, NVF-B, NVF-C, and NVF-D are 6.0 g (84.4 mmol), 8.4 g (118 mmol), 12.0 g (169 mmol), and 14.4 g (203 mmol), respectively. To a solution of ethanol (68.0 g) containing the appropriate NVF mass in a 250 mL round-bottom flask, AIBN (0.241 g, 1.45 mmol), poly(VP-*co*-VA) (1.822 g), and NVEE (0.845 g, 3.91 mmol) were added. The round-bottom flask was equipped with an overhead stirrer, nitrogen supply, and a reflux condenser. The suspension was purged with N_2_ and then heated to 70 °C. The reaction was continued for 1 h at 70 °C, and the N_2_ atmosphere was maintained while being stirred vigorously. After cooling at room temperature, the dispersion was purified by repeated centrifugation/redispersion cycles in ethanol. The final dispersion in ethanol had a concentration of 30 wt %.

### EA-MAA MG Synthesis

The synthesis of EA-MAA-based MGs used seed-feed emulsion polymerization following a method reported earlier.^51^ Briefly, a mixed comonomer solution (250 g) containing EA (164.4 g, 1.64 mol), MAA (82.2 g, 0.95 mol), and DVB (3.4 g, 0.026 mol) was prepared. Seed formation was conducted using a portion of the comonomer mixture (31.5 g) after addition to water (517.5 g) containing SDS (1.8 g) and K_2_HPO_4_ (3.15 g of 7.0 wt % solution) that had been heated to 80 °C. APS (10.0 g of 2.0 wt % solution in water) was then added to the solution with mechanical stirring under a nitrogen atmosphere. After 30 min, the remaining comonomer solution was added at a constant rate of 2.4 g min^–1^. After completion of the feed, the temperature was maintained at 80 °C for a further 2.5 h. The product was extensively dialyzed against water. Vinyl functionalization of the MG was conducted by adding GMA (30.0 g, 0.20 mol) to the mechanically stirred MG dispersion (400 g, 5.0 wt %) at pH 5.0. The dispersion was heated at 50 °C for 4 h. The product was cooled in an ice bath, and unreacted GMA was removed by washing with CHCl_3_ (200 mL) in a separating funnel twice. Residual CHCl_3_ was removed by rotary evaporation at room temperature, and the vinyl-functionalized EA-MAA MG was concentrated to 10 wt %.

### Composite DXMG Gel Preparation

The DX MG(NVF-*y*)_*x*_ gels prepared in this study contain EA-MAA MGs and NVF MGs in specific proportions. The parameter *x* is the dry wt % of the NVF MG used in each sample, and *y* is the code for the NVF MG used. For example, DX MG(NVF-A)_25_ refers to a composite DX MG containing 75% EA-MAA MG and 25% NVF-A MG. The gels were prepared at a concentration of 12.0 wt % and pH 7.6. An example preparation is given for DX MG(NVF-A)_25_ gel. NVF-A MG dispersion (5.0 g, 30 wt % in ethanol) and water (15.17 g) were added under stirring to form a dispersion. Ethanol was then removed by rotary evaporation at room temperature to afford an aqueous dispersion with a concentration of 9.0 wt %. An EA-MAA MG dispersion (2.00 g, 13.56 wt %) was added to the aqueous NVF MG (1.00 g, 9.0 wt %), and the combined dispersions were subjected to vortex mixing until uniform. Aqueous NaOH solution (4.0 M, 190 μL) with TEMED solution (68 μL, 1.6 wt %) and APS solution (68 μL, 2.0 wt %) was then added to the dispersion simultaneously and mixed. The mixture rapidly formed a physical gel. The masses of all the reactants used to prepare the DX MG(NVF-*y*)_*x*_ gels examined in this study are shown in Table S2. The composite gels were formed by heating the physical gels in sealed molds at 37 °C overnight. The DX MG control (*x* = 0) was prepared using similar conditions to those discussed above with the omission of NVF.

### Physical Measurements

Potentiometric titration was conducted using a Mettler Toledo Easy Plus titrator. The titrations were performed in the presence of aqueous NaCl (0.010 M), and the titrant was aqueous NaOH solution (0.10 M). The *z*-average diameters (*d*_*z*_) of the MGs were measured using dynamic light scattering (DLS). The data were measured with a 50 mW He/Ne laser operated using a standard avalanche photodiode (APD) and 901 detection optics. This Malvern Zetasizer Nano ZS instrument comprised a 50 mW He/Ne laser, a standard APD, and 901 detection optics connected to a ZS90 correlator. The swelling behavior of the MGs was assessed by determining the particle volume swelling ratio (α_v(p)_), which was calculated using the following equation.
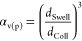
1where *d*_Swell_ is the swollen diameter and *d*_Coll_ represents the collapsed diameter. The EA-MAA MG particles were considered to be in their collapsed state at pH 4.0 in water. For NVF MG, the particles were considered to be in the collapsed state when dispersed in ethanol, which is a bad solvent for PNVF. In the case of the hydrogels, the volume swelling ratio (α_v(G)_) was calculated from the mass swelling ratio (α_m(G)_), which was measured gravimetrically, and used the polymer and water densities (ρ_p_ and ρ_w_) with the following equation.
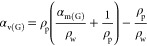
2

SEM images were obtained using a TESCAN instrument. The current and voltage used were 1.0 pA and 5.0 kV, respectively. For the pore size measurements, we used three SEM images per sample for every gel. A total of 100 pores were measured per sample. Oscillatory rheology measurements were conducted using a TA Instruments DRH3 temperature-controlled rheometer equipped with an environmental chamber. A parallel plate geometry (diameter = 20 mm) was used. For the strain–sweep data, a frequency of 1.0 Hz was used. Uniaxial compression measurements were conducted using an Instron series 3344 load frame equipped with a 100 N compression load cell. The gel samples were cylindrical, and the height and diameter were both 13 mm. Optical microscopy (OM) was conducted with an Olympus BX41 microscope. Confocal microscopy images were obtained using a Leica TCS SP8 confocal microscope. The excitation and emission wavelengths were 550 and 620 nm, respectively. The imaging experiments involving 3RAX were conducted as follows. A 3RAX solution (0.40 mM) was prepared. Then, DX MG pieces were immersed in the 3RAX solution for 3 days. After that, the stained DX MG pieces were immersed in water for 2 days, and the water was changed twice every day to remove excess 3RAX.

### Cell Viability and MTT Assay

Human NP cells were derived from IVD samples obtained with informed patient consent under ethical approval from the research ethics committee (London—Brighton & Sussex. Ref. no: 17/LO/1408 improving the understanding of IVD degeneration, diagnosis, and treatment). The cells were maintained in Dulbecco’s modified Eagle’s medium supplemented with 10% fetal bovine serum (Gibco) and antibiotic/antimycotic (Merck) at 37 °C in a humidified 5% CO_2_ atmosphere. To determine cell viability, NP cells were seeded at a density of 1 × 10^5^ onto 13 mm glass coverslips in 24-well plates (Sarstedt) and cultured overnight before the addition of toroid-shaped gels. The viability of cells was observed at 24 h versus gel-free controls by live/dead assay (Life Technologies, UK). Images were obtained with a Zeiss Axio Observer 71 fluorescent microscope. For cell metabolic activity, NP cells were seeded at a density of 5 × 10^4^ per well onto 24-well plates and allowed to adhere overnight before exposure to 10 mg of gel via 0.4 μm cell culture inserts (BD Biosciences). MTT assays (Merck) were carried out at as per the manufacturer’s instructions using a FLUOstar OMEGA plate reader.

## Results and Discussion

### Characterization of NVF and EA-MAA MGs

**Figure 1:**
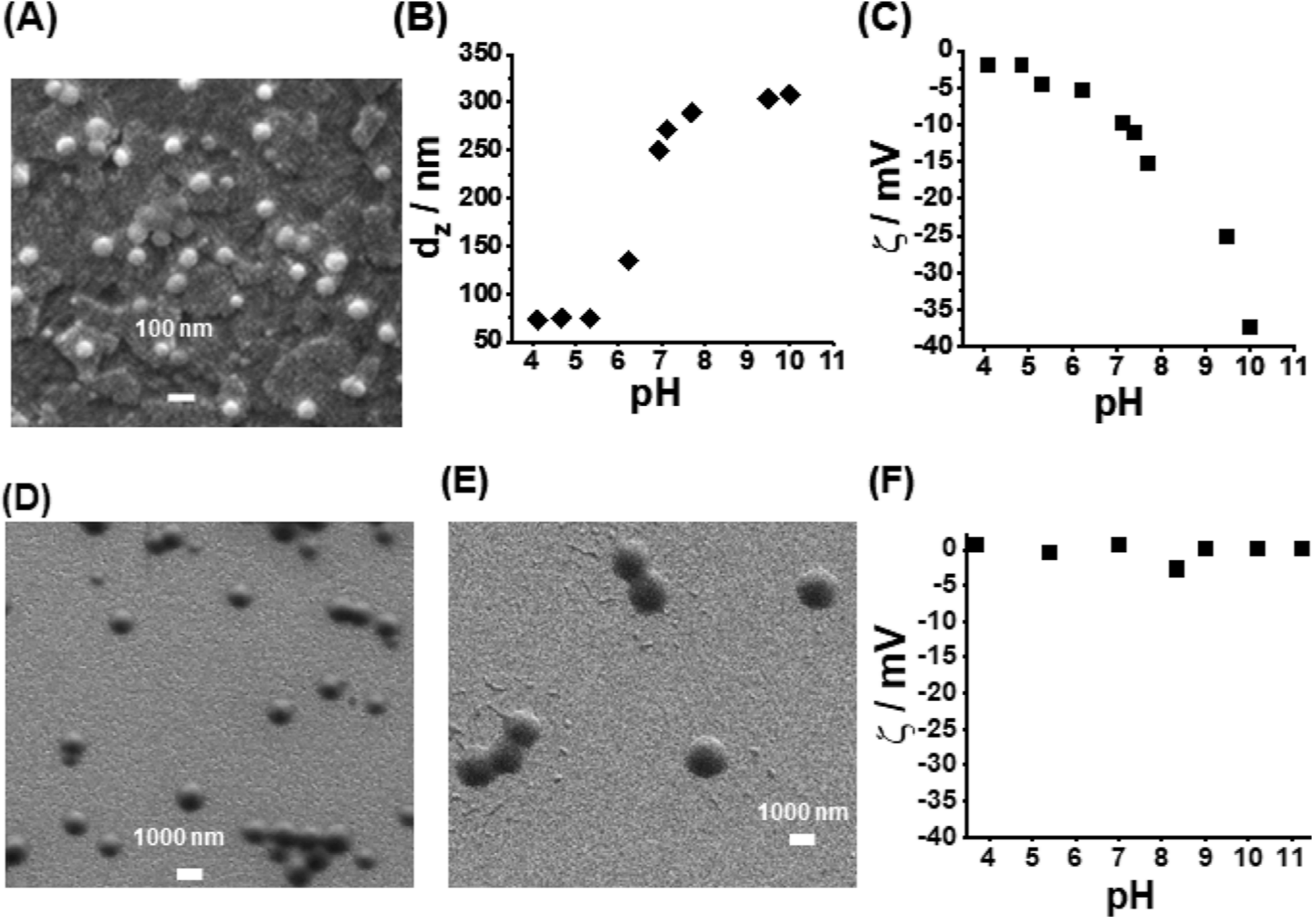
(A) SEM image of EA-MAA MGs deposited from water. The pH-responsive behavior of the (B) *z*-average diameter and (C) zeta potential are shown for the EA-MAA MGs. SEM images for (D) NVF-A MGs and (E) NVF-D MGs deposited from ethanol. Note the difference in scale bars in (A) compared to (D,E). (F) Variation of zeta potential with pH for the NVF-A MGs in water.

The EA-MAA MG building block of the DX MGs had a composition of PEA_0.63_–MAA_0.36_–(MAA-GMA)_0.02_–DVB_0.01_ (Scheme 1) and an apparent p*K*_a_ value of 6.8 based on titration data (Figure S2). The number-average diameter for the EA-MAA MGs from SEM (Figure 1A) is 72 nm. The EA-MAA MGs are strongly pH-responsive (Figure 1B), and their *d*_*z*_ values increased from 75 to 303 nm as the pH increased from 4.7 to 10, respectively. This is due to the intersegment electrostatic repulsion from the RCOO^–^ groups. The increase in *d*_*z*_ value corresponds to a high α_v(p)_ value of 66 according to eq 1. The zeta potential data (Figure 1C) confirm that the EA-MAA MGs become increasingly negatively charged as the pH increases.

**Figure 2:**
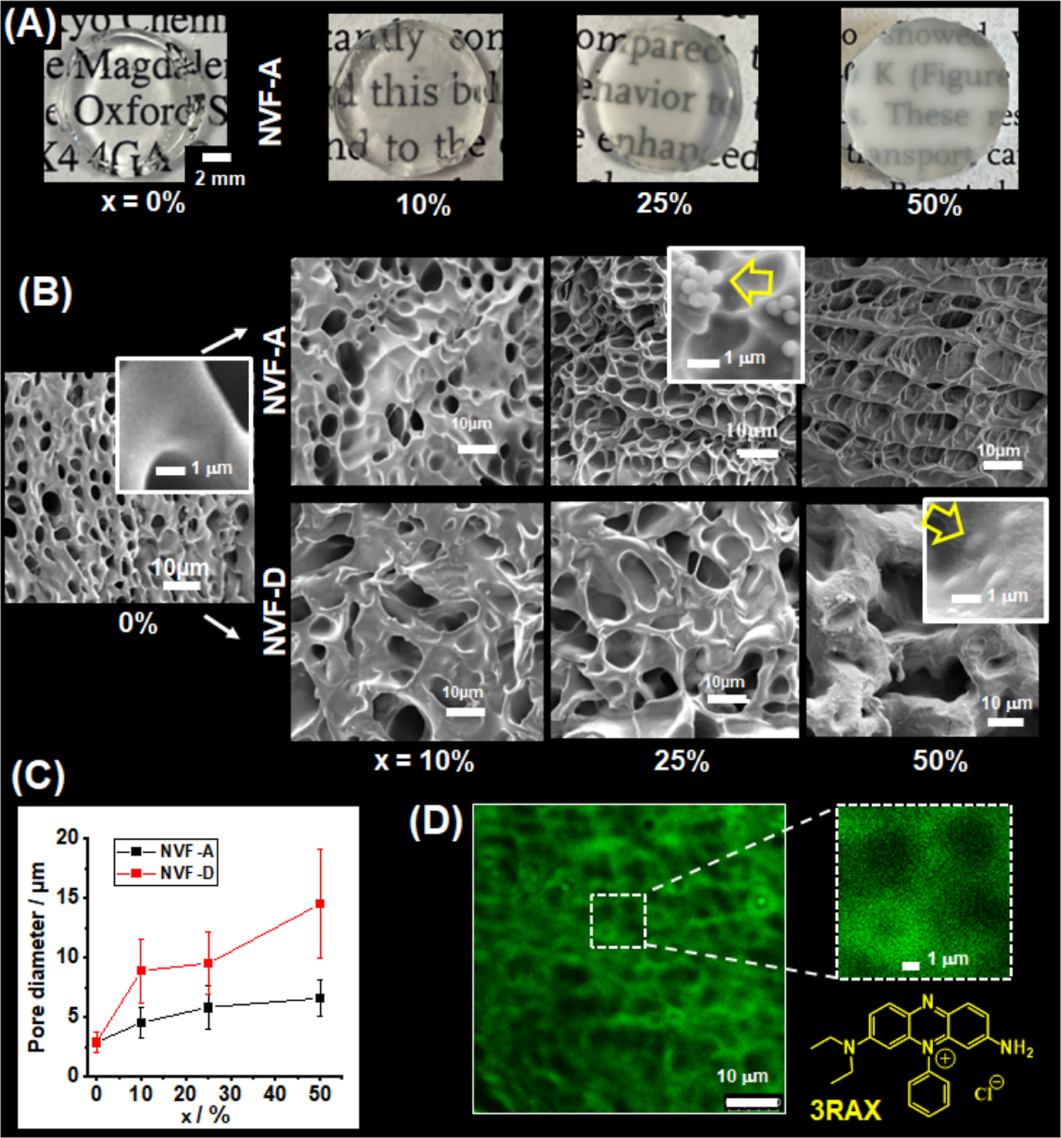
(A) Photographs of DX MG(NVF-A)_*x*_ gels. The values for *x* are shown. (B) SEM images of freeze-dried DX MG(NVF-A)_*x*_ and DX MG(NVF-D)_*x*_ gel samples. The yellow arrows in the insets show NVF MGs. (C) Average pore diameters from the SEM data shown in (B). (D) Confocal microscopy image for DX MG(NVF-D)_10_ gel in water that had been pre-stained with methylene violet (3RAX). The structure of the latter is shown.

Four NVF MG systems were prepared with increasing diameter (designated as NVF-A to NVF-D). All of these particles had diameters of ∼1000 nm or larger, which meant that OM was well suited for measuring their size. The OM images for the NVF MGs dispersed in ethanol (bad solvent) and water (good solvent) are shown in Figure S3. The number-average diameter measured by OM for the as-prepared NVF MGs in ethanol (*d*_OM_) increased linearly from 960 nm (for NVF-A) to 2060 nm (for NVF-D) with the NVF concentration used during particle synthesis (see Figure S4). We focused on the smallest (NVF-A) and largest (NVF-D) MGs in this study because the former gave the best composite gel mechanical properties and the latter were most easily resolved using OM (see below). The SEM images for these MGs are shown in Figure 1D,E. (The SEM images for the NVF-B and NVF-C particles are shown in Figure S5.) The number-average diameters from SEM for the (collapsed) NVF-A and NVF-D MG particles are 794 and 1892 nm, respectively. Hence, the NVF-A and NVF-D MGs are ∼ factors of 11 and 26 larger, respectively, compared to the EA-MAA MGs. The OM images for these MGs dispersed in water (Figure S3) show that the α_v(p)_ values for NVF-A and NVF-D are 2.7 and 3.0, respectively (Table S1). The relatively low α_v(p)_ values compared to that for the EA-MAA MGs (α_v(p)_ = 66) is due to the non-ionic nature of the NVF MGs. This is supported by the zeta potentials for the NVF-A MGs, which are not dependent on pH (Figure 2F) or significantly different from zero. Hence, the NVF MGs have negligible charge and are not pH responsive. This conclusion is supported by the DLS data measured as a function of pH (Figure S6) for the NVF-A MGs in water. The mean of the *d*_*z*_ values for the latter was 1150 ± 114 nm over the pH range of 4–11.

### DX MG(NVF-*y*)_*x*_ Composite Gel Morphologies and Mechanical Properties

In a one-step process, we mixed the vinyl-functionalized EA-MAA MGs and the NVF-A or NVF-D MGs in the presence of APS and TEMED (Scheme 1, stage 1). The pH increased to 7.6, which is greater than the apparent p*K*_a_ of the EA-MAA MGs (6.8) and triggered particle swelling causing immediate physical gel formation (Stage 2). The neighboring EA-MAA MGs then formed covalent inter-MG crosslinks and produced a chemical gel at 37 °C (Stage 3). This process encapsulated the relatively large NVF MG particles within the interlinked DX MG network. The NVF MG-free DX MG (*x* = 0%) had high transparency as shown in Figure 2A. As the NVF-A content increased (*x* increased), the gel transmittance decreased. This is confirmed by the UV–visible transmittance spectra for the gels shown in Figure S7A. We quantify the visible light transmission using the average visible transmittance (AVT), which is the average value of the transmittance over the visible wavelength range (380–760 nm). The AVT decreased from 67.7% for the pure gel (*x* = 0%) to 49.4% when *x* = 50% as shown by Figure S7B. This trend is due to the difference in the refractive index values (and hence light scattering ability) between the EA-MAA MGs and NVF MGs in the composite gel. The EA-MAA MGs are strongly swollen compared to the NVF MGs as judged by their respective α_v(p)_ values (Table S1). A lower α_v(p)_ implies a higher polymer volume fraction and a higher refractive index. Consequently, the NVF MGs scatter light when dispersed in the EA-MAA MG matrix. The relatively large size of the NVF-A MGs also contributes to light scattering.

The SEM images of freeze-dried DX MG(NVF-*y*)_*x*_ gels are shown in Figure 2B. All of the gels form porous morphologies after freeze-drying. The pores in freeze-dried hydrogels are caused by water freezing.^52^ The insets of Figure 2B for the NVF-A (*x* = 25%) and NVF-D (*x* = 50%)-based composite gels show individual NVF-A and NVF-D MGs, respectively. The average gel pore size increases as the NVF-A and NVF-D MG concentrations increase, as shown in Figure 2C. (Pore size distributions are shown in Figure S8.) An increase in the pore size of freeze-dried DX MGs is an indirect indication of a decrease in the stiffness of the matrix.^51^ It follows that the stiffness (and hence modulus) of these gels decreases as the covalent interlinked EA-MAA MG network is gradually replaced by a non-MG network forming NVF MGs. Water contact angle measurements were conducted for the freeze-dried gels (Figure S9) and revealed that inclusion of the NVF-A MGs caused the contact angle to decrease from 116° for the DX MG to 70° for the DX MG(NVF-A)_50_ gel. Hence, inclusion of NVF MGs into the DX MG matrix increased the hydrophilicity of the gel network.

To examine the morphology of the gels in the hydrated state, we stained a DX MG(NVF-D)_10_ gel with methylene violet (3RAX) to enhance contrast. The structure of 3RAX is shown in Figure 2D. This cationic dye preferentially absorbed to the negatively charged EA-MAA MGs. A representative OM image (Figure S10) of the gel showed black spheres with a diameter of ∼2.0–3.0 μm, which are due to the NVF-D MGs that did not absorb the dye. Confocal microscopy (Figure 2D) also showed black spheres in the same size range as observed by OM. These data show that the gel morphology consists of relatively large NVF-D MGs encapsulated by smaller (sub-micrometer) interconnected EA-MAA MGs. Furthermore, the confocal data suggest that the NVF-D MGs are well dispersed within the DX MG matrix. Such dispersion is a particular advantage of using binary MG mixtures as injectable gels and should ensure the uniformity of mechanical properties throughout the composite gel.

Preliminary strain–sweep rheology measurements were conducted using DX MG(NVF-A)_25_ and DX MG(NVF-D)_25_ gels (see Figure S11). The shear modulus (*G*′) values for these gels are 6.49 and 2.23 kPa, respectively. The strain (γ) at the point of intersection of the *G*′ and loss modulus (*G*″) curves [i.e., tan δ = (*G*″/*G*′) = 1] corresponds to the fluidization strain^53^ (γ*). The γ* value decreases as crosslinking increases^54^ and is a measure of ductility. The values of γ* for the DX MG(NVF-A)_25_ and DX MG(NVF-D)_25_ gels are 207 and 60%, respectively. The DX MG(NVF-A)_25_ gel is considered to have better mechanical properties than the DX MG(NVF-D)_25_ gel because it has a much higher γ* value and hence ductility. We focus on the DX MG(NVF-A)_*x*_ gels for the remainder of the study because the ultimate application of IVD repair requires an injectable gel that can withstand shear.

**Figure 3:**
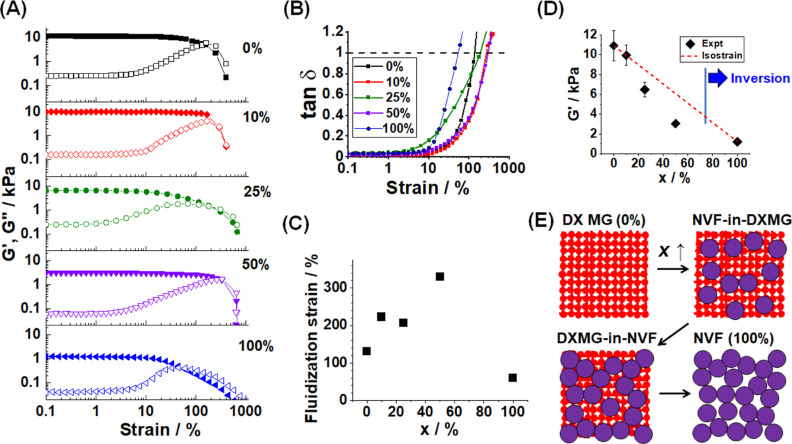
(A) Strain–sweep rheology data for DX MG(NVF-A)_*x*_ gels prepared with different values of *x* (shown). *G*′ and *G*″ are the storage and loss modulus values, respectively. (B) Variation of tan δ (= *G*″/*G*′) with strain. (C) Variation of the fluidization strain (strain at which tan δ = 1.0) with *x*. (D) *G*′ data (at 1% strain) fitted to the isostrain model (see text). (E) Conceptual model for the arrangements of EA-MAA and NVF MGs within DX MG(NVF-*y*)_*x*_ gels as *x* increases.

We therefore investigated the mechanical properties of the DX MG(NVF-A)_*x*_ gel series from Figure 2A using dynamic rheology. Strain–sweep data are shown in Figure 3A. When these data are plotted in terms of reduced *G*′ and *G*″ values vs reduced strain, the presence of strain softening with a weak strain overshoot is evident (Figure S12), and the systems show type III behavior as discussed in the Supporting Information. It is evident from Figure 3A that the *G*′ values are independent of strain at low strain values and then decrease and intersect *G*″. Consequently, tan δ is very low at small strains and then increases passing through 1.0 when γ* is reached (Figure 3B). The γ* values are shown in Figure 3C as a function of *x*. The γ* values increase from 130% for DX MG (*x* = 0%) to 330% for DX MG(NVF-A)_50_. The γ* value then decreases to 60% for the *x* = 100% NVF-A physical gel. The latter system does not contain a covalent DX MG network to distribute strain. The present data confirm that inclusion of NVF-A into the DX MGs increases ductility, which is important for potential application because the IVD is subjected to dynamic strain in vivo*.*^55^ The ductility increase for the DX MG(NVF-A)_*x*_ gels is ascribed to inclusion of the swollen, deformable NVF-A MGs which dissipate strain energy.

The value of *G*′ for the DX MG(NVF-A)_*x*_ gels decreases strongly with increasing *x*, as shown Figure 3D. Hence, inclusion of NVF-A in the DX MG network causes the gel to become less stiff. These data confirm the conjecture regarding gel stiffness inferred from the pore size variation observed in Figure 2C. The *G*′ vs *x* data shown in Figure 3D were fitted using a modified version of the isostrain model.^56^ The latter considers the behavior of soft particles dispersed in a stiff matrix.
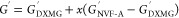
3

For eq 3, *G*′_DXMG_ and *G*′_NVF-A_ are the modulus values of the DX MG matrix and the dispersed NVF-A MGs, respectively. The value for *x* is the wt % of NVF-A in the DX MG(NVF-A)_*x*_ gels. The isostrain model assumes that the strain experienced by the relatively soft (dispersed) particles is the same as that of the stiffer (DX MG) matrix. We assume that *G*′_NVF-A_ is the same as that measured for the *x* = 100% NVF-A physical gel. The fit to eq 3 is good for three of the five data points. However, the fit overestimated the *G′* values for the *x* = 25 and 50% gels.

Based on the above results, we propose a conceptual model to explain the mechanical behavior for the DX MG(NVF-A)_*x*_ gels, which is depicted in Figure 3E. At low *x* values, the composite gel has isolated NVF-A MGs dispersed within a continuous covalently interlinked EA-MAA MG network. These gels comprise a NVF-in-(DX MG) composite gel. Provided that NVF-A MGs are perfectly dispersed, the mechanical properties follow the isostrain model of eq 3. However, as *x* increases, percolation of the NVF-A MGs occurs, leading to regions where the DX MG network is isolated and elastically ineffective. This prevents those crosslinks from contributing to the DXMG matrix modulus, which causes a lower *G*′ compared to that predicted by eq 3. As the *x* content increases further, the proportion of the DX MG network that is isolated increases, which increases the difference between the calculated and experimental *G*′ values in Figure 3D. When the *x* > 50%, phase inversion occurs and a DX MG-in-(NVF-A) gel forms. This system has a continuous NVF-A physical gel phase containing dispersed islands of DX MG and has a low *G*′. Such gels redisperse in water and were not suitable for further study. Accordingly, the remainder of this study focused on DX MG(NVF-A)_*x*_ gels prepared using *x* less than or equal to 50%.

**Figure 4:**
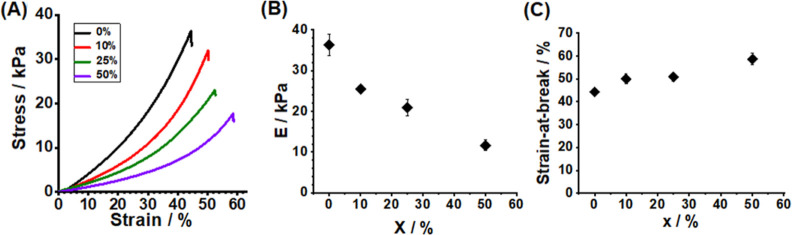
(A) Uniaxial compression stress–strain data for DX MG(NVF-A)_*x*_ gels. The variations of the (B) compression modulus and (C) strain at break with *x* are shown.

Because our ultimate target application is load support for IVD repair, uniaxial compression measurements were performed (Figure 4A). As *x* increases, the DX MG(NVF-A)_*x*_ gels become softer and more ductile, which is consistent with the rheology data from Figure 3A. Figure 4B shows that the compressive modulus decreases from 36.3 ± 2.7 to 11.6 ± 1.3 kPa as *x* increases from 0 to 50%. Cloyd et al. reported that the unconfined compressive modulus for a human NP is 5.39 ± 2.56 kPa.^27^ An injectable gel should have a stiffness that is as close as possible to that of the natural tissue to prevent mechanical damage during loading.^42^ Accordingly, the *x* = 50% composite gel has the closest modulus (11.6 ± 1.3 kPa) to a human NP in the present study. This result demonstrates the advantage of including NVF-A MGs within DX MGs to tune the gel. Furthermore, the strain at break (Figure 4C) increases from 44.2% (for *x* = 0) to 58.7% (for *x* = 50%), which confirms that inclusion of NVF-A MGs dissipates strain.

### pH-Responsive Gel Swelling

**Figure 5:**
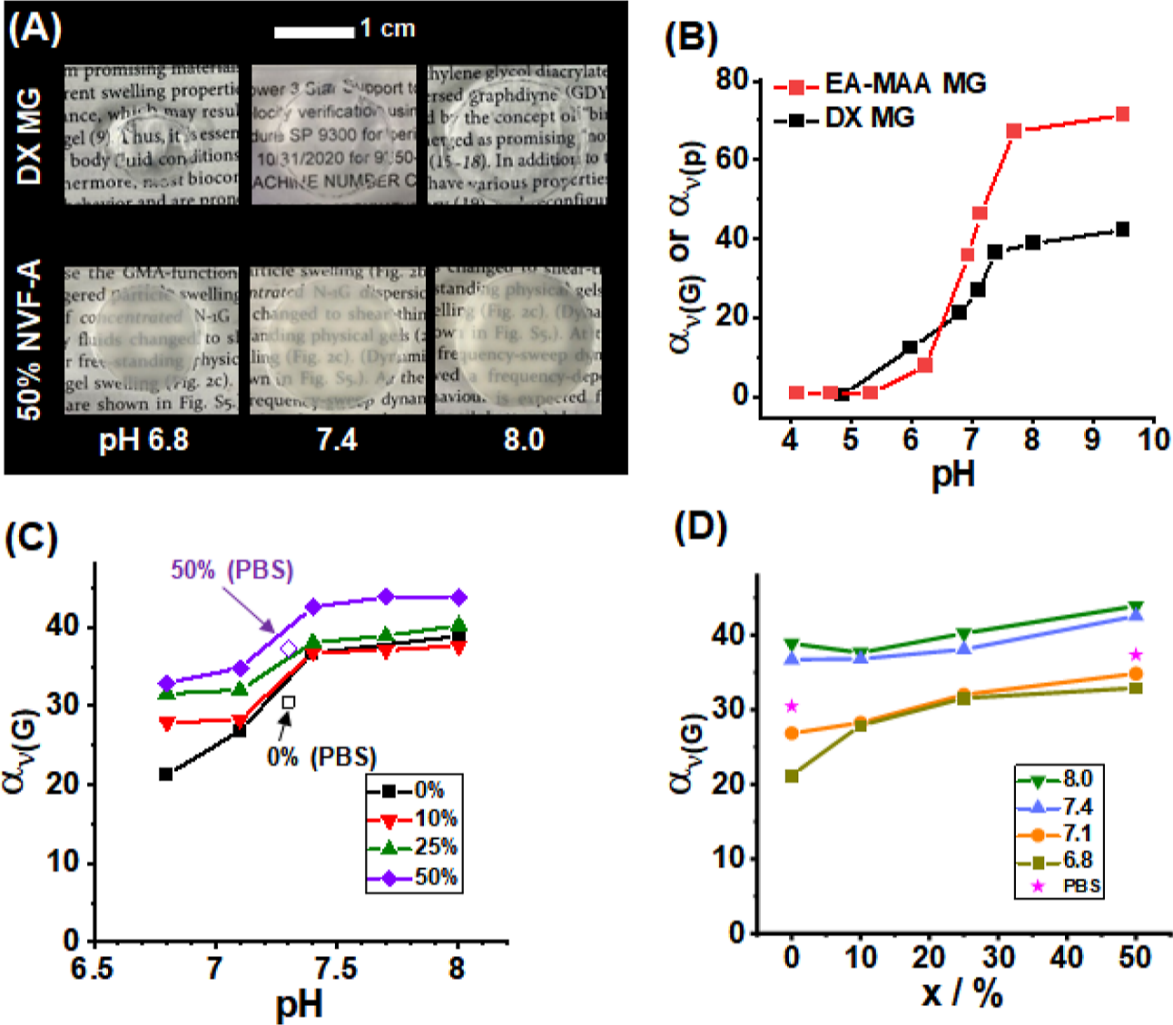
(A) Photographs of DX MG and DX MG(NVF-A)_50_ gel disks swollen at different pH values. (B) Comparison of pH-dependent DX MG swelling ratios and EA-MAA MG particle swelling ratios. (C) pH dependence of the swelling ratios for the DX MG(NVF-A)_*x*_ gels. The values for *x* are shown. Data measured using PBS are shown for comparison. (D) Data from (C) replotted to show the effects of *x* at fixed pH values. The swelling data for all of the gels were measured after 4 days of immersion in the buffer solutions.

We investigated the pH-responsive properties of the DX MG(NVF-A)_*x*_ gels. Figure 5A shows the photographs of DX MG and DX MG(NVF-A)_50_ gels swollen at pH 6.8, 7.4, and 8.0. The gels reached equilibrium swelling within 4 days of immersion in the buffer solutions as shown by the time-dependent swelling data in Figure S13. The pH-responsiveness of α_ν(G)_ for DX MG is compared to the swelling ratio of the parent EA-MAA MG particles (α_ν(p)_) in Figure 5B. The DX MGs swell less than the MGs at pH values greater than 7.0 because of additional crosslinking from inter-MG crosslinks within the DX MGs.

The pH-dependent α_ν(G)_ values for all of the DX MG(NVF-A)_*x*_ gels studied are shown in Figure 5C. The extent of pH-dependent swelling change between pH 6.8 and 8.0 decreases with increasing NVF-A content. This is because the overall pH-responsive component of the composite gel decreases as the EA-MAA MGs are replaced by non-pH-responsive NVF-A MGs. Data are also shown for the *x* = 0 and 50% gels measured in PBS for comparison. These α_ν(G)_ values are slightly lower than for the other solutions, which is due to the higher ionic strength of PBS (0.15 M) compared to the buffers (0.10 M). The data from Figure 5C are replotted in Figure 5D to illustrate the effects of *x* on the swelling ratio at fixed pH values. It is the lower pH values in the vicinity of the EA-MAA MG p*K*_a_ of 6.8 that the greatest α_ν(G)_ increase with NVF-A content occurs. This trend is due to the fact that the EA-MAA MG particles are not fully swollen at pH values of 6.8 and 7.1, and so the effect from varying *x* for the uncharged NVF MGs is more pronounced. At the higher pH values (7.4 and 8.0), where the EA-MAA MGs are swollen, the additional swelling afforded by the NVF-A MGs is also evident (but less pronounced) as *x* increases. Hence, including 50% NVF-A MGs in the DX MGs resulted in greater swelling (and hence water content) at physiological pH compared to the 0% DX MG.

### DX MG(NVF-A)_50_ as an Injectable Gel

**Figure 6:**
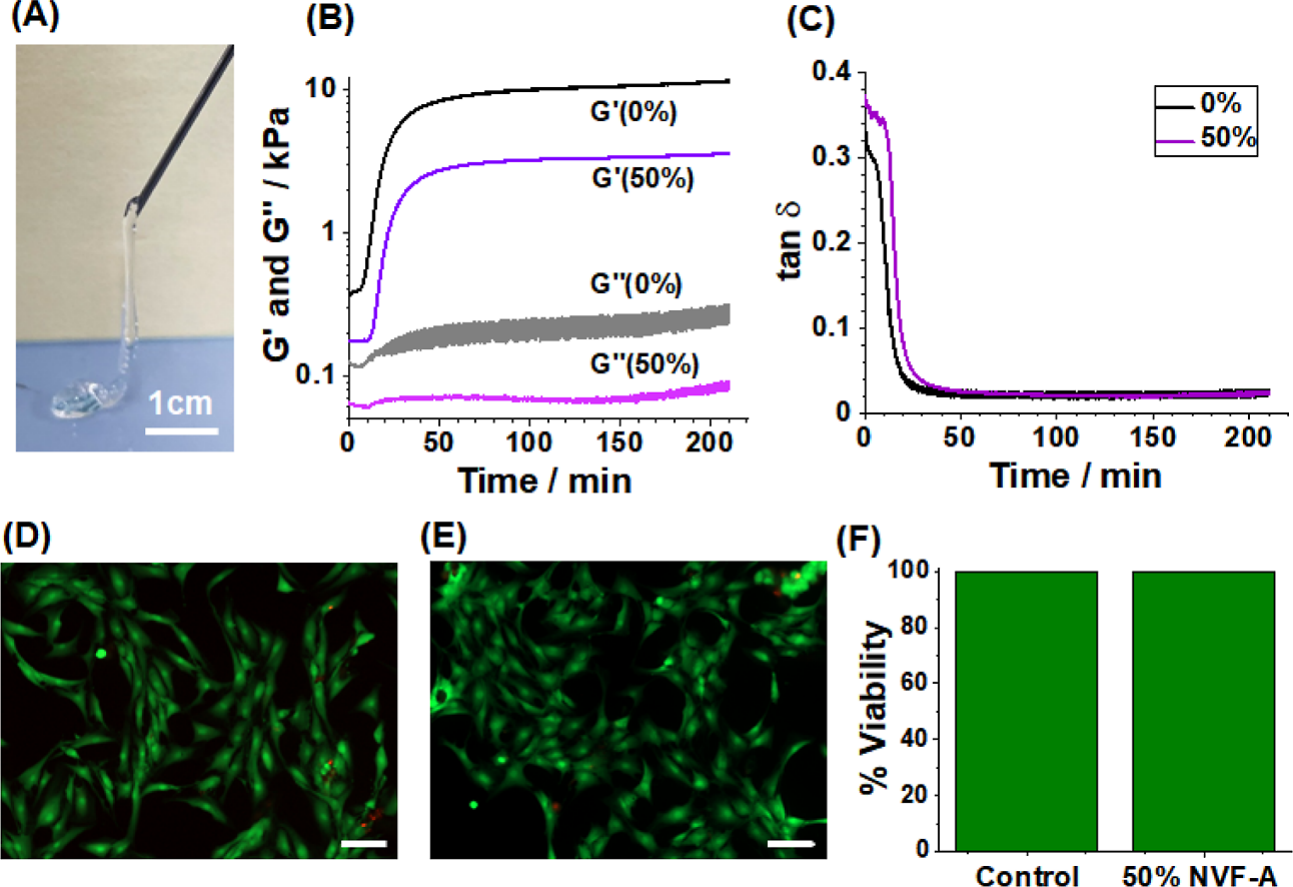
(A) Photograph of the physical gel precursor for DX MG(NVF-A)_50_ being injected through a syringe needle. (B) Variation of *G*′ and *G*″ with time for DX MG and DX MG(NVF-A)_50_ gels. (C) Variation of tan δ with time from the data shown in (B). The measurements for (B,C) were performed using 1.0% strain, 1.0 Hz, and 37 °C. Live/Dead images for a (D) gel-free control and the (E) DX MG(NVF-A)_50_ gel using NP cells. The scale bars for (D,E) are 100 μm. (F) MTT assay results for a control and the DX MG(NVF-A)_50_ gel. The data in (D–F) were obtained after 24 h.

Given the relative similarity of the modulus for DX MG(NV-A)_50_ and that for a human NP, we investigated this system as an injectable gel for potential NP augmentation. The freshly prepared precursor dispersion for DX MG(NVF-A)_50_ was injectable through a 21 G needle (Figure 6A). It was injectable prior to DX MG formation due to the shear-thinning nature of the physical gel precursor (Scheme 1, stage 2). Figure 6B shows the time-dependent changes of *G*′ and *G*″ for the for DX MG(NVF-A)_50_ and DX MG precursor dispersions measured at 37 °C. At time = 0, *G*′ exceeds *G*″, confirming that the dispersions are, indeed, gels. The initial physical gel state is advantageous for an injectable gel because it can hold its position after injection prior to curing. The data shown in Figure 6B show that within 10 min of injection, the *G*′ values begin to increase, which is due to inter-EA-MAA MG covalent linking. The gels become increasingly solid-like with time as is evidenced by the lower tan δ values (Figure 6C). Within 40 min of injection, both gels have reached 80% of their final *G*′ values and are effectively cured. A live/dead assay was conducted using NP cells for the DX MG(NVF-A)_50_ gel and the data compared to a control gel-free system. Both the gel-free control (Figure 6D) and DX MG(NVF-A)_50_ gel (Figure 6E) show many live cells after 24 h. MTT data were also obtained for the DX MG(NVF-A)_50_ gel (Figure 6F). The viability of the NP cells in the presence of the gel was 99.8%. These data show that the gels did not adversely affect the viability of NP cells under the conditions studied. Overall, these data provide support for the longer term development of injectable DX MG(NVF-A)_50_ gels for IVD augmentation.

## Conclusions

In this study, we have investigated new injectable composite DX MGs that contain uncharged micrometer-sized NVF-based MGs for the first time. The NVF MGs disperse uniformly through the DXMG matrix and thereby confer uniform mechanical properties to the gel. Including the NVF MGs provides a new method, enabling the modulus of the DX MGs to be conveniently tuned to values close to that of NP tissue while increasing the gel ductility. The composite DX MGs retain pH responsiveness and are more swollen at physiological pH than pure DX MGs. Furthermore, the precursor physical gels are injectable and reach 80% of the final modulus within 40 mins of injection. Live/dead and MTT assays indicate that DX MG(NVF-A)_50_ is not cytotoxic to NP cells. Consequently, the work provides a potentially useful approach for tuning the properties of DX MGs for IVD augmentation while retaining their swelling and load supporting properties.

## Supporting Information Available

Scheme of method for preparing PNVF MGs, ^1^H NMR, pH-titration data, OM data, SEM, and DLS data of MGs; transmittance spectra, contact angles, rheology data, and swelling ratio of DX EA(NVF-*y*)_*x*_ gels; key parameters of MGs; and materials used for DX EA(NVF-*y*)_*x*_ gels (PDF)
