# Supplementary material for: Injectable Colloidal Hydrogels of N-Vinylformamide Microgels Dispersed in Covalently Interlinked pH-Responsive Methacrylic Acid-Based Microgels
Source: Biomacromolecules. 2023 Apr 7;24(5):2173–83. doi: 10.1021/acs.biomac.3c00058 (PMC10170504; doi:10.1021/acs.biomac.3c00058)
Supplement: Supplementary file 1 — bm3c00058_si_001.pdf [file bm3c00058_si_001.pdf]

# SUPPORTING INFORMATION

# Injectable colloidal hydrogels of *N*-vinylformamide microgels dispersed in covalently interlinked pH-responsive methacrylic acid-based microgels

Xuelian Wang[a,]*, Daman J. Adlam[b], Ran Wang[a], Amal Altujjar[a], Zhenyu Jia[a], Jennifer M. Saunders[a], Judith A. Hoyland[b], Nischal Rai[a] and Brian R. Saunders[a,]*

a) School of Materials, University of Manchester, MECD Building A, The University of Manchester, Manchester, M1 7HL, U.K.

b) Division of Cell Matrix Biology and Regenerative Medicine, Stopford Building, The University of Manchester, Oxford Road, Manchester, M13 9PT, U.K.

Corresponding author:

Xuelian Wang (xuelian.wang@ postgrad.manchester.ac.uk)

Brian. R. Saunders (brian.saunders@manchester.ac.uk)

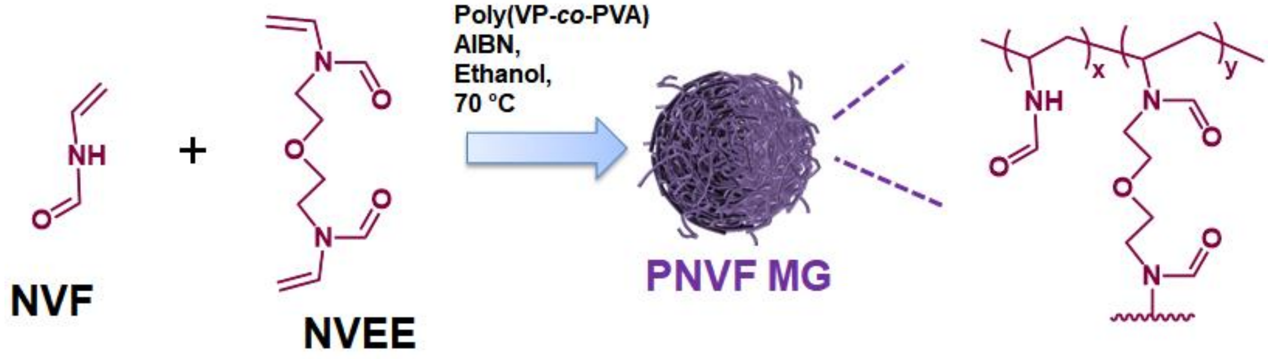


**Scheme S1.** Depiction of method used to prepare PNVF MGs.

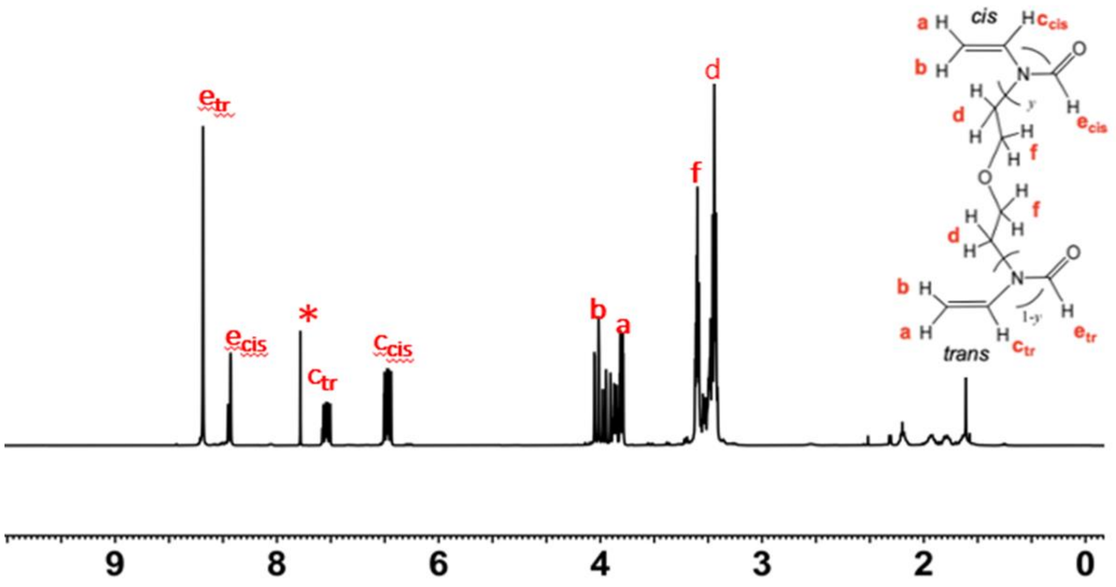


**Figure S1.** $^{1}$H NMR spectrum for NVEE. The asterisk indicates a solvent ($CDCl_3$) peak.

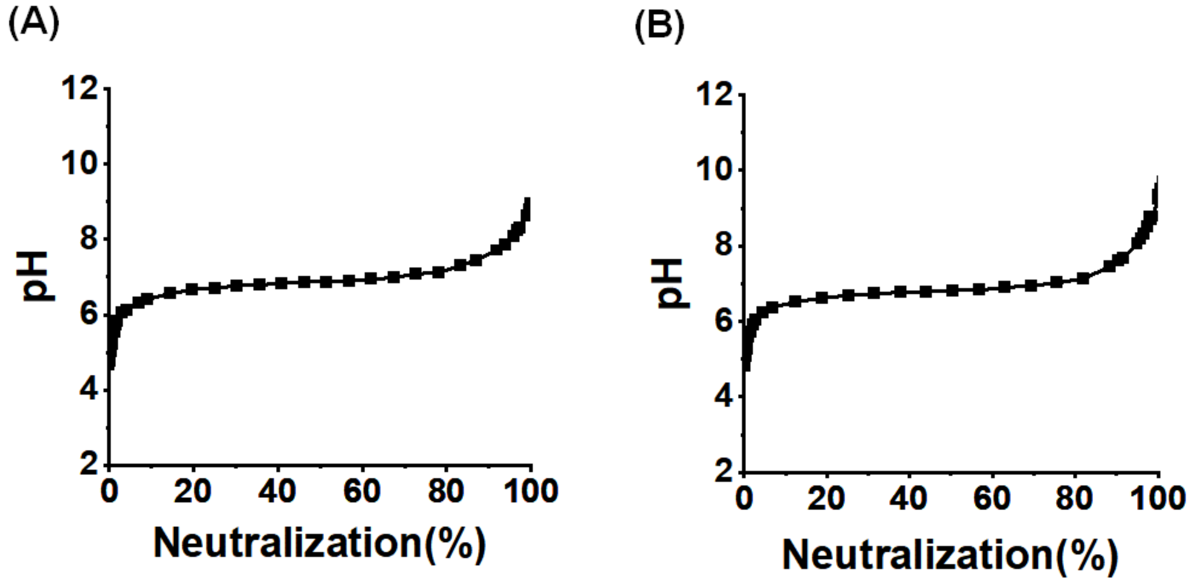


**Figure S2.** Titration data for the **(A)** pre-cursor non-GMA functionalized and **(B)** GMA-functionalized EA-MAA MGs. The MAA content before and after functionalization are 32.45 wt.% and 27.25 wt.%, respectively. The apparent p$K_a$ for these MG corresponds to the pH at which the neutralization is 50%.

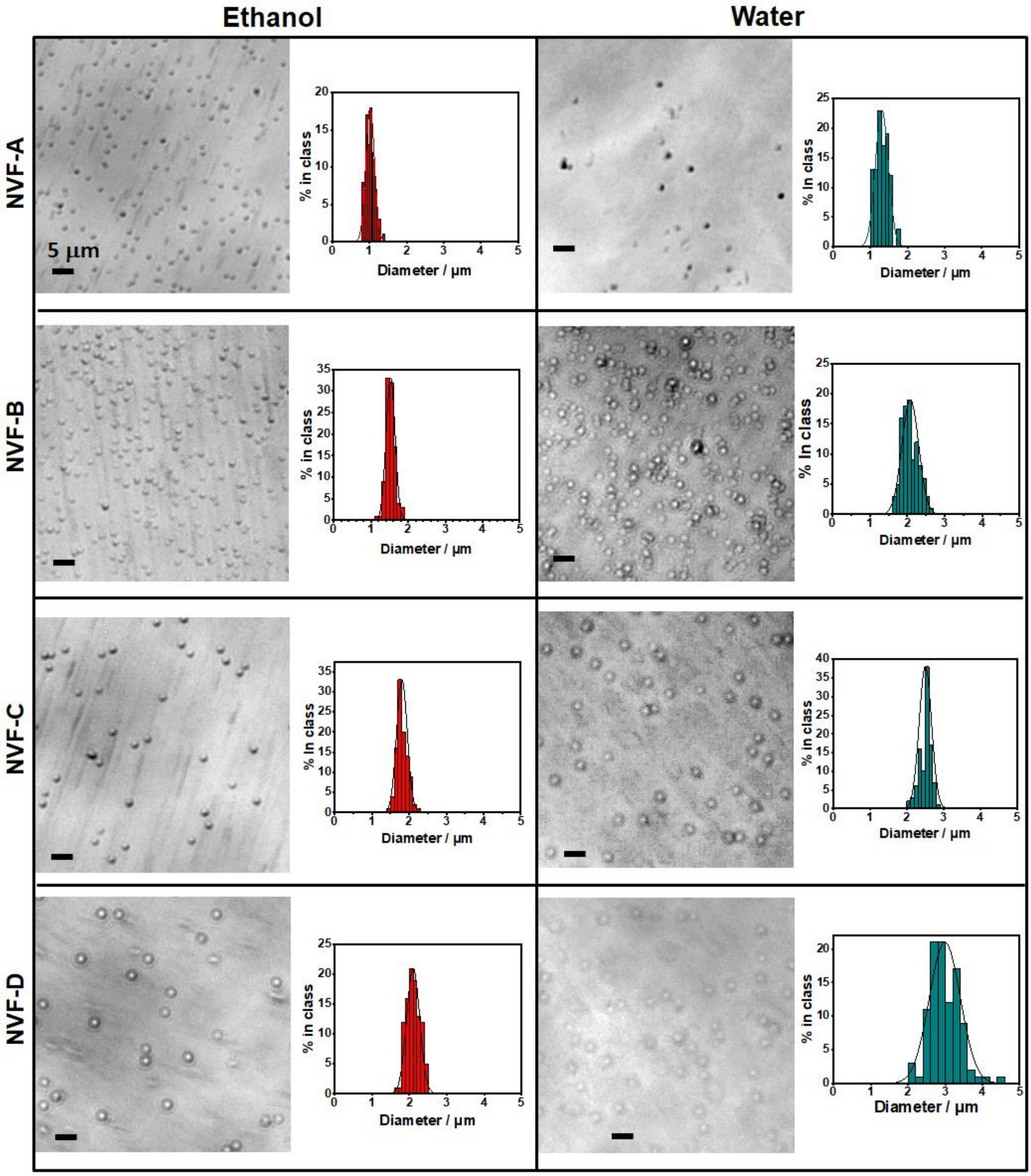


**Figure S3.** Optical micrographs of all of the NVF MG systems dispersed in ethanol (bad solvent) or water (good solvent). The size distributions are also shown.

.

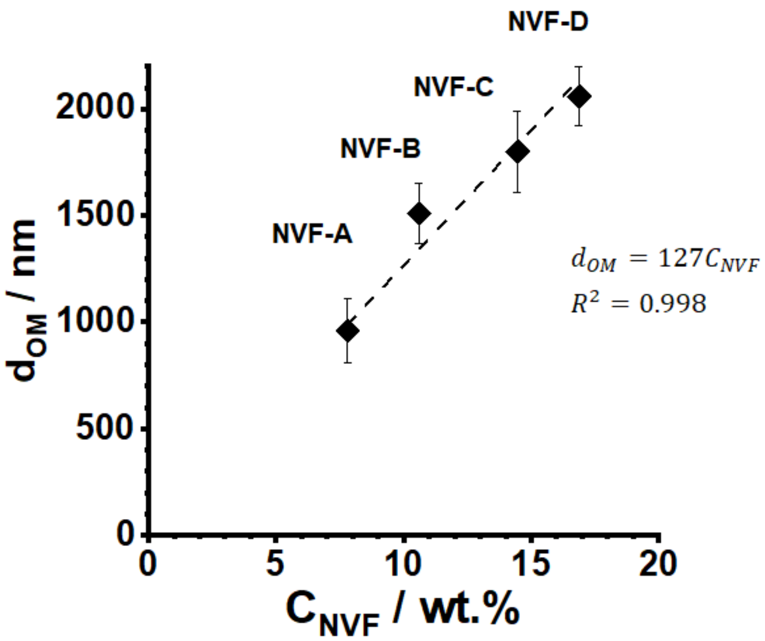


**Figure S4.** Variation of the as-made NVF MG particle diameter in ethanol measured using optical microscopy with NVF concentration used during synthesis.

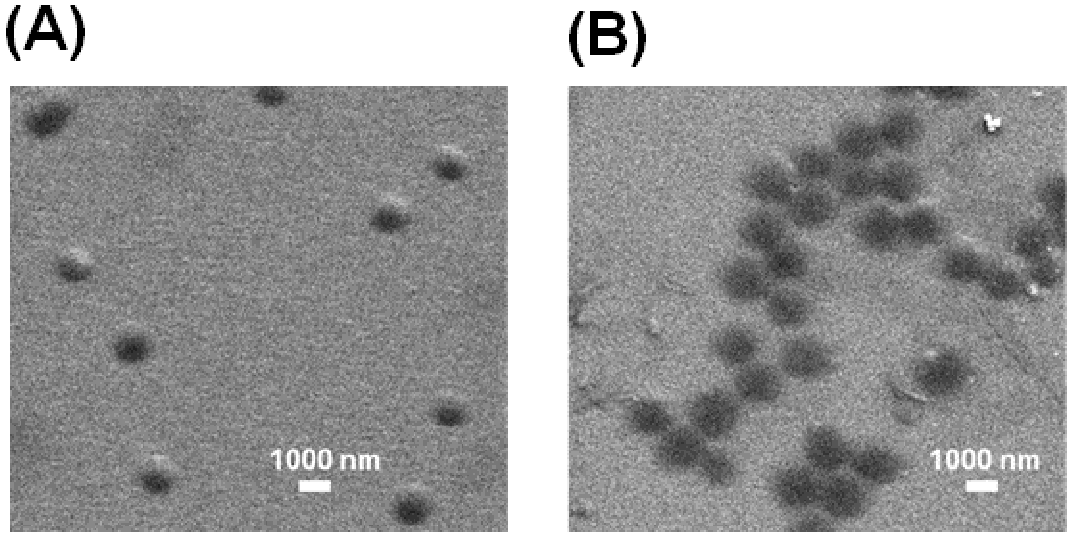


**Figure S5.** SEM images for **(A)** NVF-B and **(B)** NVF-C particles deposited from ethanol.

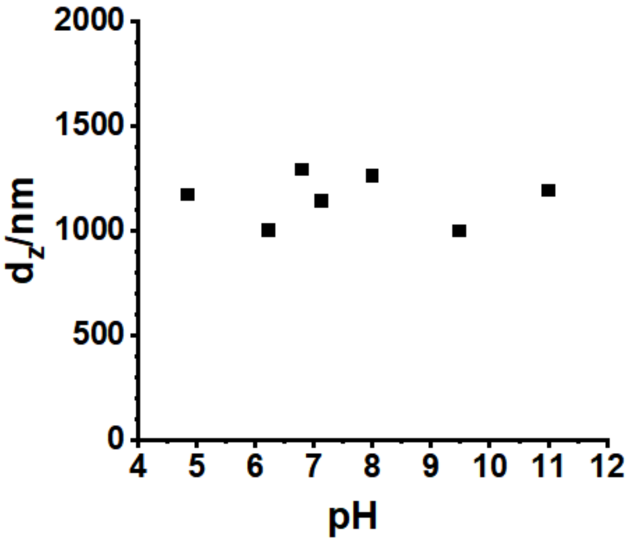


**Figure S6.** Variation of z-average diameter with pH for the NVF-A MGs in water

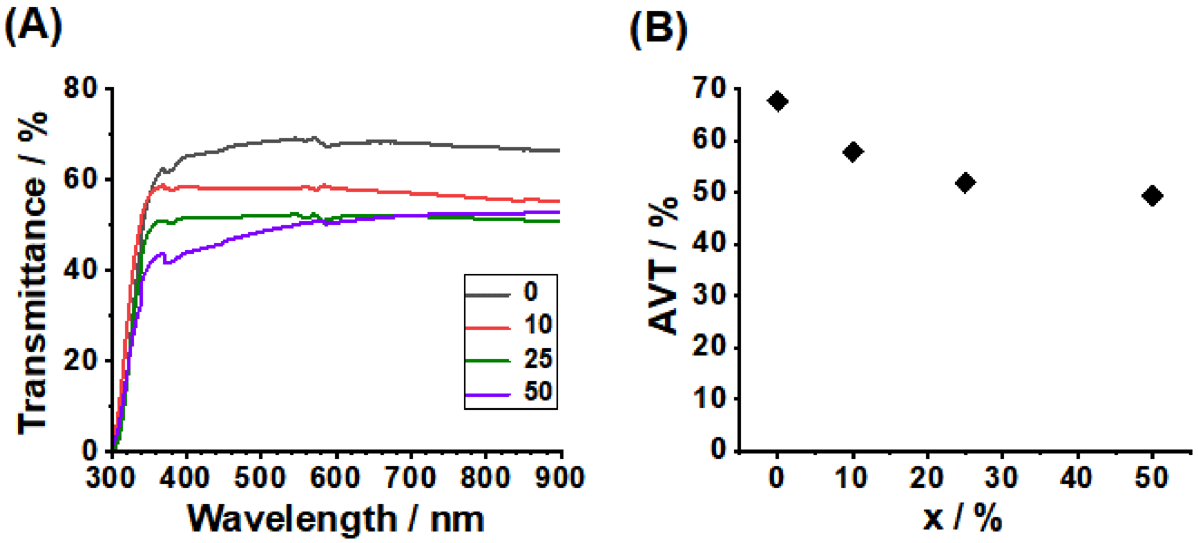


**Figure S7. (A)** Transmittance spectra for various DX MG(NVF-A)$_x$ gels and **(B)** average visible transmittance calculated over the wavelength range of 380 to 760 nm. The thickness of the gels was 2.0 mm.

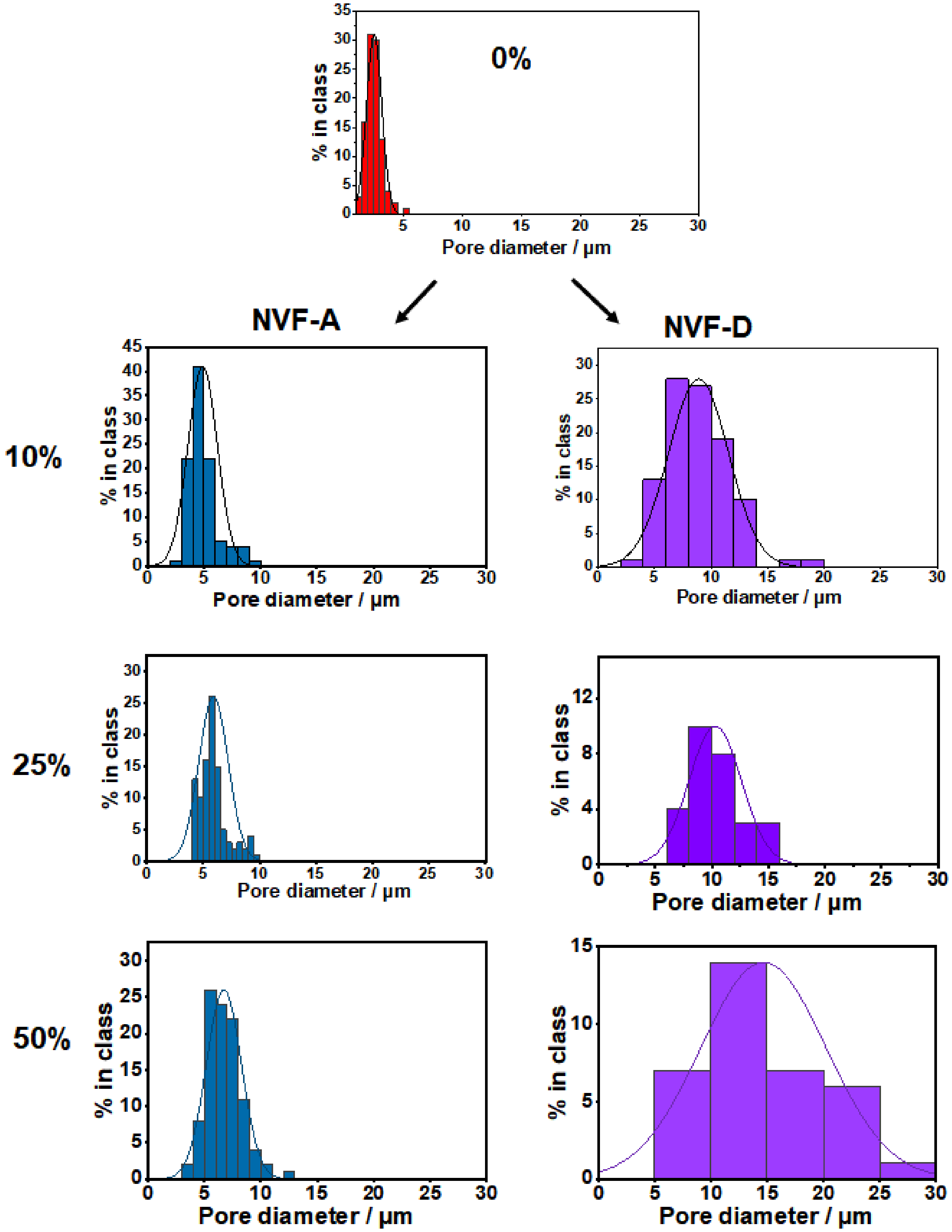


**Figure S8.** Pore size distributions for various DX MG(NVF-*y*)$_x$ gels. The values for *x* are shown. The distributions for the gels containing NVF-A and NFV-D are shown in the left- and right-hand columns, respectively.

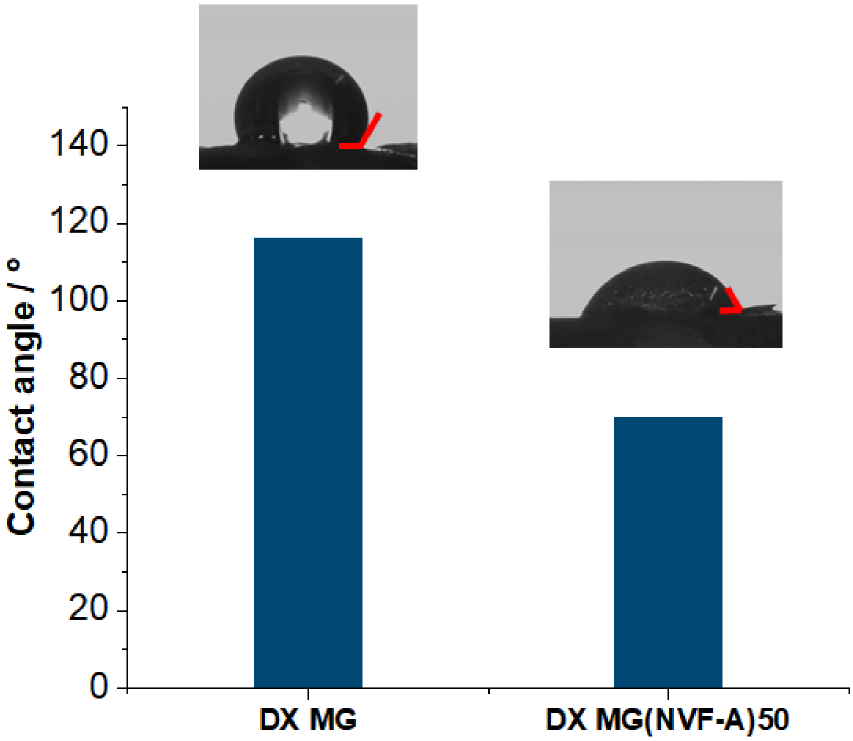


**Figure S9.** Water contact angles for a freeze-dried **(A)** DX MG gel and a **(B)** DX MG(NVF-A)$_{50}$ gel.

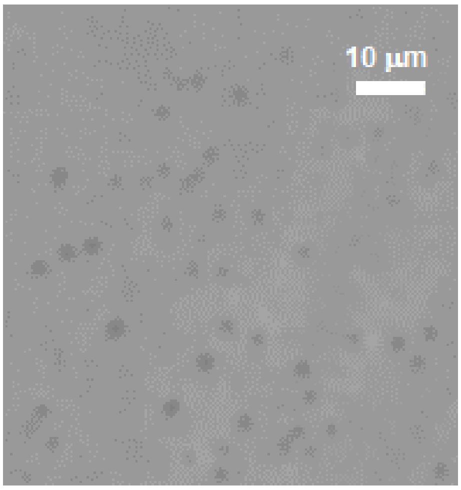


**Figure S10.** Optical micrograph of DX MG(NVF-D)$_{10}$ gel that had been pre-stained with the cationic dye, methylene violet (3RAX).

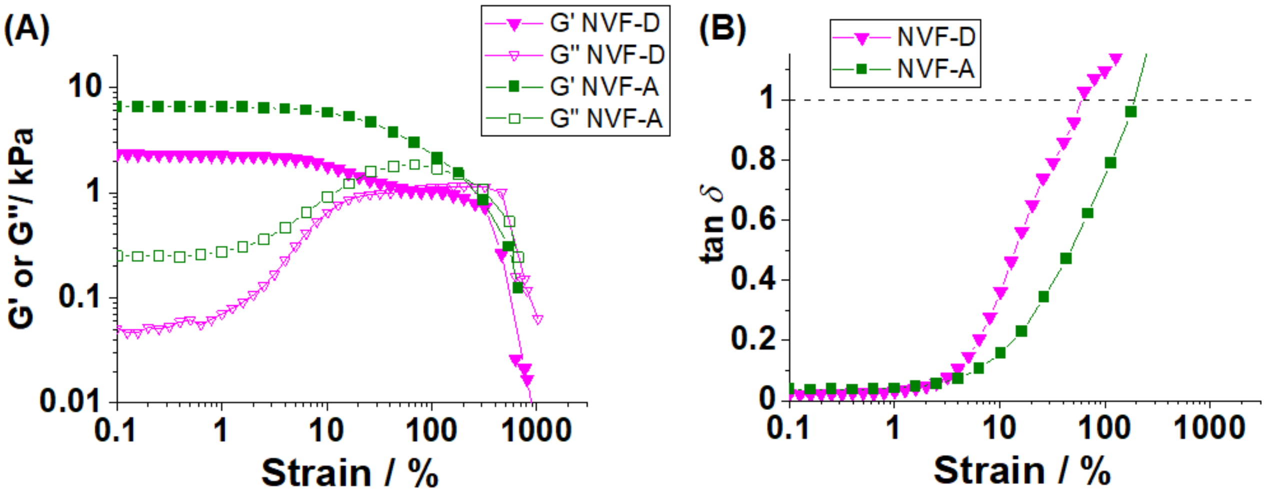


**Figure S11. (A)** Variation of the storage modulus (*G'*) and loss modulus (*G''*) with strain for DX MG(NVF-A)$_{25}$ and DX MG(NVF-D)$_{25}$ gels. **(B)** Variation of tan $\delta$ (= $G''/G'$) with strain using the data from (A).

**Considering the rheology data in terms of Type III (weak strain overshoot) behavior**

Strain-sweep rheology data can provide additional mechanistic information when plotted in terms of reduced storage modulus (*G'*/*G'(0)*) and reduced loss modulus (*G"/G"(0)*) vs. reduced shear strain ($\gamma/\gamma_c$).[1, 2] The values for *G'(0)* and *G"(0)* are the respective moduli at low strain (here, $\gamma$ = 0.1%). The value for $\gamma_c$ is the critical strain value and corresponds to the strain at which *G"* began to increase. The reduced data and the $\gamma_c$ values are shown in Figure S12. All of these data show strain-softening (*G'* decreasing) and a weak strain overshoot (*G"* increasing then decreasing). Hence, a maximum in *G"* only is observed and our systems are identified as Type III according to the large amplitude oscillatory shear behaviors identified by Kyun et al.[2] Type III behavior is the result of a structural change that occurs at larger strain amplitudes. However, there is no universal mechanism for the cause of the strain overshoot and the cause is system dependent. The present gels contain a combination of covalent interlinked EA-MAA MGs and NVF MGs that are in the physically gelled state. We speculate that the overshoot for the DX MG(NVF-A) gels (*x* = 10%, 25% and 50%) has contributions from breaking of inter-EA-MAA MG covalent bonds and cage-breaking for the physically gelled NVF MG domains.

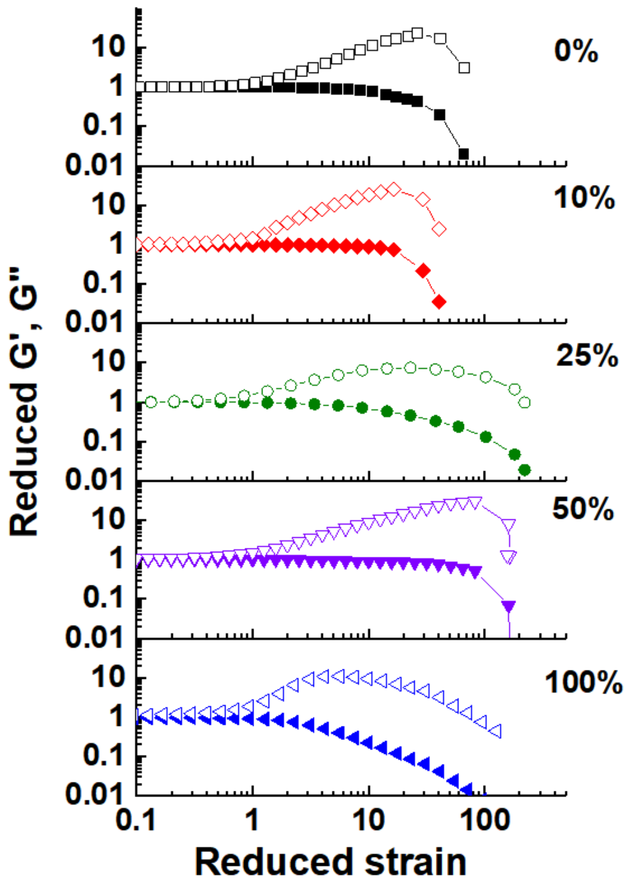


| %NVF | $\gamma_c$ / % |
|---|---|
| 0 | 6 |
| 10 | 10 |
| 25 | 3 |
| 50 | 4 |
| 100 | 8 |

**Figure S12.** Reduced strain-sweep rheology data for DX MG(NVF-A) gels. The values for *x* are shown and the data are taken from Figure 3. Strain overshoot behavior is indicated by the *G''* maxima. The $\gamma_c$ values used for the reduced strain are shown in the table.

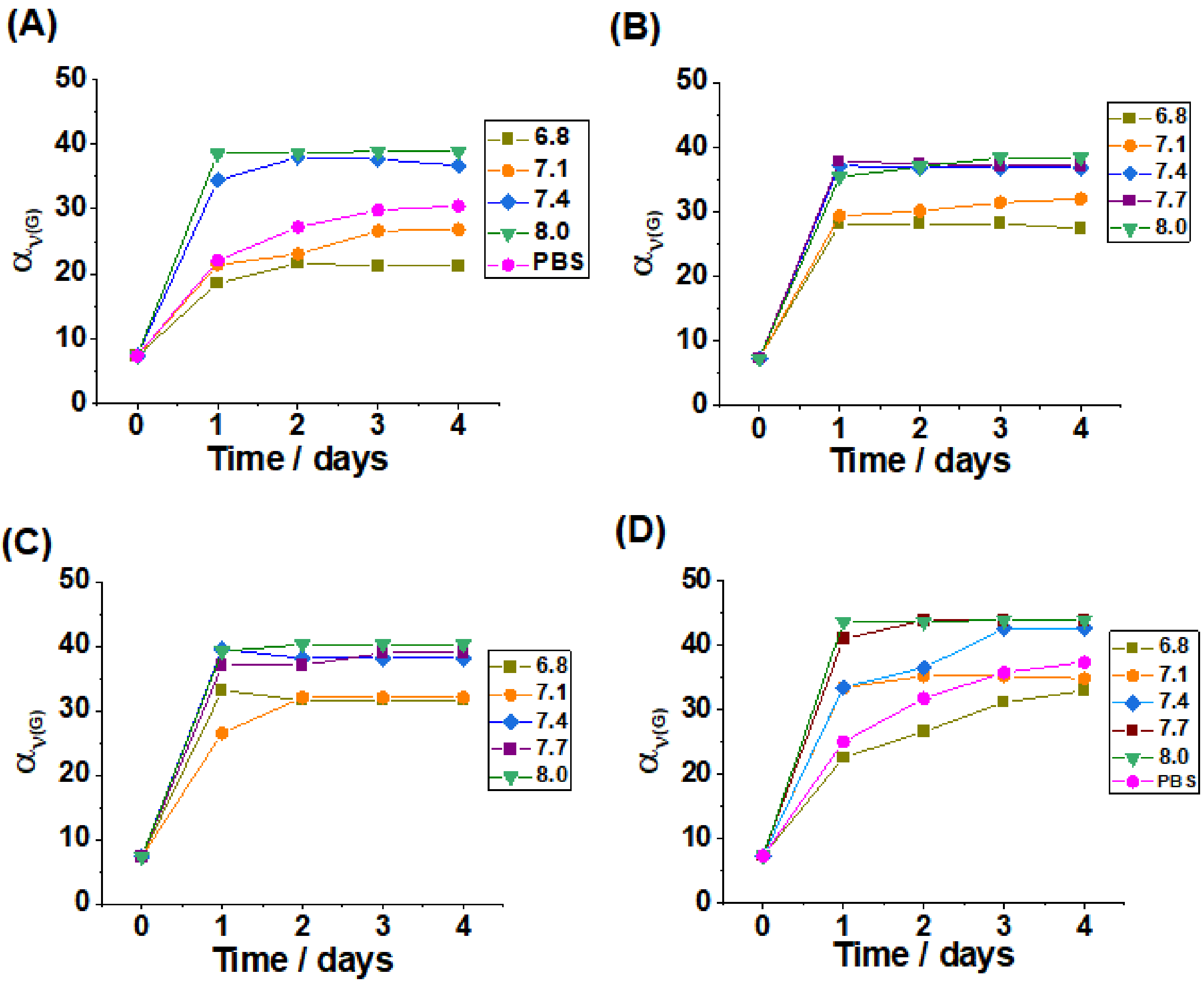


**Figure S13.** Swelling ratio changes with time for **(A)** DX MG(NVF-A)$_x$ gels prepared using *x* values of **(A)** 0%, **(B)**, 10%, **(C)**, 25% and **(D)** 50%.

**Table S1.** Key parameters for the MGs studied

| MG code | $d_{SEM}$ [a] / nm | $d_{OM(coll)}$ [b] / nm | $d_{OM(swollen}$ [b] / nm | $\alpha_{v(p)}$ [c] | ζ [d] / mV |
|---|---|---|---|---|---|
| EA-MAA | 72 ± 7 | 75 [e] | 303 [f] | 66 | -20.2 |
| NVF-A | 794 ±10 | 960 ± 150 | 1342 ± 146 | 2.7 | - |
| NVF-B | 1400 ± 160 | 1510 ± 140 | 2070 ± 230 | 2.6 | - |
| NVF-C | 1710 ± 200 | 1800 ± 190 | 2760 ± 27 | 3.6 | - |
| NVF-D | 1892 ±160 | 2060 ± 140 | 2970 ± 300 | 3.0 | 0.6 |

[a] Number-average diameter measured by SEM.

[b] Diameter measured using optical microscopy in ethanol (collapsed) or water (swollen).

[c] Particle volume swelling ratio calculated according to equation 1 at pH 7.7.

[d] Zeta potential. The data for PEA-MAA and PNVF-D were measured at pH = 7.4.

[e] Obtained using DLS at pH = 4.7

[f] Obtained using DLS at pH = 10

**Table S2.** Materials used for preparing DX EA(NVF-*y*)$_x$ gels

| $x$ | EA-MAA MG[a] / g | NVF MG (wt.%) / g | Water added / g | APS / μL | TEMED / μL | NaOH / μL |
|---|---|---|---|---|---|---|
| 0 | 1.77 | - | 0.23 | 60 | 60 | 168 |
| 10 | 2.00 | 0.50 (6.00) | - | 68 | 68 | 190 |
| 25 | 2.00 | 1.00 (9.00) | - | 68 | 68 | 190 |
| 50 | 1.74 | 2.36 (10.00) | - | 59 | 59 | 165 |

[a] The concentration of the stock EA-MAA MG used was 13.56 wt.%

## References

1. Ben Messaoud, G.; Le Griel, P.; Hermida-Merino, D.; Roelants, S. L. K. W.; Soetaert, W.; Stevens, C. V.; Baccile, N., pH-Controlled Self-Assembled Fibrillar Network Hydrogels: Evidence of Kinetic Control of the Mechanical Properties. *Chemistry of Materials* **2019,** *31*, 4817-4830.

2. Hyun, K.; Wilhelm, M.; Klein, C. O.; Cho, K. S.; Nam, J. G.; Ahn, K. H.; Lee, S. J.; Ewoldt, R. H.; McKinley, G. H., A review of nonlinear oscillatory shear tests: Analysis and application of large amplitude oscillatory shear (LAOS). *Progr. Polym. Sci.* **2011,** *36*, 1697-1753.
